# Extending Validity of the Bacterial Cell Cycle Model through Thymine Limitation: A Personal View

**DOI:** 10.3390/life13040906

**Published:** 2023-03-29

**Authors:** Arieh Zaritsky

**Affiliations:** Faculty of Natural Sciences, Life Sciences Department, Ben-Gurion University of the Negev, Kiryat Bergman, HaShalom St. 1, Be’er-Sheva 8410501, Israel; ariehzar@gmail.com; Tel.: +972-54-595-5670

**Keywords:** bacterial physiology, division cycle, cell dimensions, nucleoid complexity, replication position, eclipse

## Abstract

The contemporary view of bacterial physiology was established in 1958 at the “Copenhagen School”, culminating a decade later in a detailed description of the cell cycle based on four parameters. This model has been subsequently supported by numerous studies, nicknamed BCD (The Bacterial Cell-Cycle Dogma). It readily explains, quantitatively, the coupling between chromosome replication and cell division, size and DNA content. An important derivative is the number of replication positions *n*, the ratio between the time *C* to complete a round of replication and the cell mass doubling time *τ*; the former is constant at any temperature and the latter is determined by the medium composition. Changes in cell width *W* are highly correlated to *n* through the equation for so-called nucleoid complexity *NC* (=(2*^n^* − 1)/(ln2 × *n*)), the amount of DNA per *terC* (i.e., chromosome) in genome equivalents. The narrow range of potential *n* can be dramatically extended using the method of thymine limitation of thymine-requiring mutants, which allows a more rigorous testing of the hypothesis that the nucleoid structure is the primary source of the signal that determines *W* during cell division. How this putative signal is relayed from the nucleoid to the divisome is still highly enigmatic. The aim of this *Opinion* article is to suggest the possibility of a new signaling function for nucleoid DNA.

**Motto-1: *Look; don’t touch* (science principle; attributed to Ole Maaløe [1]).**

**Motto-2: *It makes sense to try clarifying ideas that emerge even if they cannot be tested right away* [2].**

## 1. Brief Historical Highlights

### 1.1. Pioneering Bacterial Physiology

The conceptual process leading to an understanding of the bacterial cell cycle was hampered by the previously known eukaryotic cycle [G_1_-S-G_2_-M(-G_0_)] because there was an expectation that the cycle would resemble that of eukaryotes. The usually circular bacterial chromosome replicates bidirectionally from a single origin *oriC*, and the unanticipated, surprising reports of reinitiating replication prior to the completion of the previous round at *terC* [3,4] revolutionized the field.

Major ideas in quantitative microbial physiology [5] were established in the so-called “Copenhagen School” led by Maalϕe [2]. The back-to-back pioneering publications from this laboratory, coauthored with Schaechter and Kjeldgaard [6,7], opened the field of bacterial physiology. The soon-after groundbreaking studies of Hanawalt [8], manipulating the “immunity” to thymine-less death (TLD) [9] and using autoradiography, discovered two distinct, seemingly independent stages involved in DNA replication, initiation and elongation.

### 1.2. The Central Dogma of the Bacterial Cell Cycle (BCD)

The seminal, extensive series of Helmstetter’s studies with *Escherichia coli* B/r during the 1960s [1] culminated with the understanding of the basic properties of the relationships between chromosome replication and cell division [10]. The ultimate quantitative description of the bacterial cell cycle includes only four parameters that can define the cell’s physiological state (ignoring the variabilities in populations). At a constant temperature under steady-state exponential growth [11], (i) doubling time *τ* depends on nutritional conditions [6]. The other three, all related to chromosome replication, are relatively constant at a wide range of *τ*s (between ~20 and 70 min) irrespective of the medium composition: (ii) strain-dependent mass per *oriC* when replication initiation occurs, *Mi* [10,12]; (iii) the time taken for a round of replication to be completed, *C*; and (iv) the time between replication-termination and cell division, *D*. In slow growth, when (*C* + *D*) < *τ*, another temporal parameter (albeit not independent) sometimes appears, the time *B* (=*τ* − (*C* + *D*)) between cell birth (upon splitting its mother) and subsequent replication initiation. This description, which was quantitively consistent with the results obtained at the time, has been repeatedly confirmed by numerous investigations during the following decades and hence may be termed as the Central Dogma of The Bacterial Cell Division Cycle (BCD) [13].

### 1.3. Average Cell Size and Chromosomal DNA Content

Applying Powell’s age distribution function f(*a*) (= (ln2/2) × 2^−*a*^), where 0 ≤ *a* ≤ 1 [14], the BCD generates, convincingly and realistically so, the average cell size and DNA content, respectively: <M> (= *Mi* × 2^(*C*+*D*)/*τ*^) [12] and <G> (= (*τ*/*C*ln2) × (2^(*C*+*D*)/*τ*^ − 2*^D^*^/*τ*^)) [10]. Two important parameters were thus derived from BCD: “set number” (*C* + *D*)/*τ*) is “the number of generations between the start of a round and the division at the end of that round” [10], and “the number of replication positions per chromosome” *n* (=*C*/*τ*), defined earlier [15] as “the position of a set of equivalent [simultaneously initiating] replication points on a chromosome. Replication with more than one position is called ‘multi-forked’ and replication with less than one position implies the presence of a resting period”. Both quantities describe the chromosomal state; the former relates to the whole cell and the latter to the chromosome itself, i.e., to its unique terminus *terC*. The simple parameter *n* turned out to be key to understand the cell’s physiological state as well; by avoiding the value of *D*, the molecular mechanism of which is still enigmatic (and see in Section 3.3), the amount of DNA per chromosome in genome equivalents *NC* (=(2*^n^* − 1)/(ln2 × *n*)) [15] and the DNA/mass ratio (i.e., “DNA concentration”) in the cell (*G*/*M*)_c_ (=(1 − 2^−n^)/(*Mi* × *n* × ln2)) [16] can be evaluated.

### 1.4. Cell Dimensions

**Figure 1:**
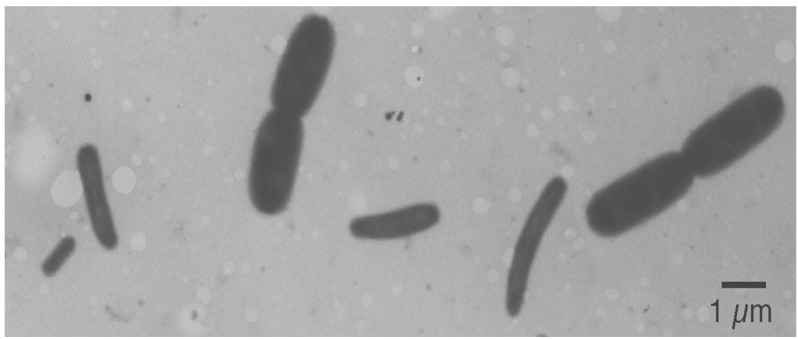
Electron micrograph (taken by Conrad Woldringh) of a mixture of two *E. coli* B/r cultures grown with doubling times *τ* = 22 min (the bigger cells) and *τ* = 150 min (the smaller cells).

A cylindrical (rod-shaped) cell such as *E. coli* grows via elongation, with a hardly discernible change in width *W* during the cell cycle under slow growth rate [17], likely due to systematic variation of “…the internal osmotic pressure…decreased during elongation and increasing again during constriction”. Unpredictably, during faster growth (shorter *τ*s) in richer media, the cells are also wider ([6,18], Figure 1, and see details below, in Table 1). With Bob Pritchard, we were the first to suggest a connection of *W*-change with the parameters of the BCD [19,20], more directly and rigorously so in [21]. The idea prevailing in the 1970s and 1980s, that cell length-growth is bi-linear, coupled to devoted, discrete envelope-growth sites, was inspired by the then-popular, so-called replicon model [22]. With the knowledge that cell mass and volume grow exponentially, *W* would passively be determined by the active extension of both, leaving no degree of freedom for the mode of its determination. Soon after the idea of “wall growth zones” was precluded experimentally [23], an alternative hypothesis was presented [24,25], that *W* is “…actively determined by the amount of DNA packed in an individual chromosome”, in which case the cell elongates by default at a rate that depends on the other two to preserve a constant mean buoyant cell density [26]. This idea was prompted by our observations [27] that during a nutritional shift up cells elongate temporarily faster than before, overshooting their final steady-state length, and that the new polar caps are the first to widen, resulting in “pear-shaped” cells, before stabilizing the new steady-state dimensions. Thus, a presumed primary signal for both, cell division and *W* determination occurs simultaneously during the action of the divisome in concerted temporal and spatial processes that must be coupled to the nucleoid segregation as well. This notion has been strengthened by our detailed studies with thymine limitation, as described below (at Section 4).

The coefficient of variation CV of the ratio *W*/*NC* is 2.9 and 4.4% in *S. typhimurium* and *E. coli*, respectively. Such small CV values for a relationship between two independently measured, seemingly unrelated parameters are rare in biological systems hence re-enforce the suggestion that they are not fortuitous.

## 2. Dissociation between Rates

The dissociation between the rates of replication *C*^−1^ and of mass growth *τ*^−1^ [10] readily explained many then-puzzling observations such as that cells are larger at richer media supporting faster growth [6] and “rate maintenance” of divisions during (*C* + *D*) min following a nutritional shift-up transition [7]. The near-constant values of *Mi*, *C* and *D* leaves little freedom to manipulate the cell cycle except *τ* through medium composition. Conditional lethal mutants in numerous, indispensable genes involved in fixing these parameters enhanced our understanding of the biochemical pathways involved in DNA replication and cell division. For example, modulating expression levels of *dnaA*-*ts* mutants at intermediate temperatures clarified the mechanism of replication initiation [30]. Such mutants are, however, usually pleiotropic, which affects other pathways and hence often blurs the picture.

As soon as this dissociation was fully understood [10], I arrived at Leicester University’s Genetics department to study for a PhD (1969–1971). My mentor Bob (Robert H) Pritchard (1930–2015) [31] immediately realized that the dissociation has two sides, and that some consequences of results in the then-current studies utilizing thymine-requiring mutants to follow DNA replication were often flawed due to the varying concentrations of thymine [T] supplied. To minimize expending radioactive material (commonly ^3^H- or ^14^C-labeled), very low [T]s have often been exploited at high specific radioactivities (Cu/gr). I was set to quantitatively test the hypothesis that, under such conditions, the value of *C* depends on [T]; in a couple of years, we succeeded to extend the validity of this dissociation by elongating *C* up to about three-fold without any noticeable change in *τ* (in identical medium) simply by modulating the supply of thymine to *thyA* mutants of *E. coli* [16,32,33,34,35]. Thus, the mean number of “replication positions” *n*, limited in Thy+ strains to ~2 due to the minimum *τ*_min_ of 20 min (whereas *C* is constant at 40 min), could only be increased by lengthening *C*. The least physiologically disturbing way to overcome this limit seems to be by reducing the externally supplied [T]. 

### The Thymine Limitation Tool

Thymine is the only base solely incorporated into DNA but is not used as such in Thy+ strains; their immediate metabolite for DNA synthesis, T-dRib-P-P-P is made from U-dRib-P in a dedicated pathway through a couple of phosphorylations of the resultant T-dRib-P [19,34], indicating that a specific permease for thymine has never evolved. In *thyA* mutants (with an inactive thymidylate synthetase), T-dRib-P is also exclusively exploited for DNA synthesis, but here it is synthesized via a bypass salvage pathway, using supplied thymine entering the cell by diffusion alone [19,34]. Intracellular [T_i_] is therefore related to the externally supplied [T]. Hence, *n* (=*C*/*τ*) can be manipulated in two ways, by varying *C* at a constant *τ* in *thyA* mutants or varying *τ* at a constant *C* in Thy+ strains. The systematic manipulation of both (which has never yet rigorously studied, to the best of my knowledge) is anticipated to dramatically affect *NC* [20] (later defined as “Nucleoid Complexity” [34]; see below for explicit definition also in Table 1, [28], and Equation (6) at [25]).

## 3. Repercussions

### 3.1. Valuable Uses of Thymine Limitation

Validating the hypothesis that *C* inversely varies with [T] by several means [16,32,33] opened the way to discover additional aspects of cell physiology at a wider range of *n* exploiting the powerful thymine limitation leverage (mostly summarized and referenced in [34]). Several attributes were studied by scientists in laboratories from all around the world, e.g., the bidirectionality of chromosome replication, kinetics of mutagenicity, control of plasmid replication, and localizing replication forks by SeqA foci distribution. Some studies were performed as follow-up at Leicester after my departure: the dependence of constitutive gene output at different DNA concentrations and relative gene dosages, dependence of *D* on *C*, and changes in cell dimensions and shape [36]. The latter two were also among the subjects that I pursued at Ben-Gurion University of the Negev (BGU) during 50 years of my tenure there, being naturally attracted by the highly effective tool of thymine limitation, mostly in cooperation with colleagues and trainees at home and abroad (see below and in [35]).

### 3.2. The Eclipse

My first years at BGU reflected several subjects related to my studies at Leicester—particularly two that are still at the heart of yet-unsolved questions: lack of a steady state in terms of cell dimensions during fast growth under thymine limitation [20], and the relationship between cell width *W* and BCD parameters [21]. An explanation of the former emerged in results of the long-term inhibition of DNA replication, envisioned as [33]: “It is postulated that this second replication position (20), which was ready to initiate when thymine was restored, remained “stacked” until the previous one had traversed the presumed minimal distance away from the origin of replication. This hypothesis should not be elaborated further, but should serve as a working hypothesis to be tested by direct means” (and see [2] Motto-2). The existence of a minimal distance possible between two successive replisomes, later termed “Eclipse” [34], was confirmed by several laboratories over-expressing *dnaA* (summed up and discussed in [37]). With a long *C*, it takes more time for the replisome to reach this distance; if this time is longer than *τ*, the real start of replication occurs at a larger cell mass than the typical *M_i_* for the said strain, and hence this delay is cumulative [37]. It reaches a maximum before branching, as does *NC*, readily explaining, at least qualitatively, the lack of steady-state cell dimensions as observed [20] under such conditions (short *τ* in rich media and long *C* at low [T]s); this is fully consistent with the concept that *W* is related to *NC* [28].

### 3.3. Dependence of D on C: Contradicting and Enigmatic Results

This dependency under thymine limitation was investigated by employing several methods, with contradictory results: (i) in my hands [38,39], using the “division-rate maintenance” phenomenon [7], when *C* was longer (at lower [T]), apparent “*D*” increased proportionately, with a relationship of [38] “*D*” = 0.83*C* − 16. On the other hand (ii), Meacock and Pritchard [40], using the “baby machine” method [1,10] and calculating from measurements of cell composition [16] (average size, DNA content and DNA/mass ratio), arrived at the opposite conclusion, that “*D*” was rather shortened upon the lengthening of *C*. Their main conclusion, however, that “the time of cell division is determined by termination…” is obvious. Option (i) seems more realistic and makes more sense because at longer *C* the cells are wider with a larger circumference hence require longer time to assemble the divisome and complete the division process, leading to a longer *D*, but then, of course, the results of the more extensive baby machine and cell composition studies [40] must be differently explained. A possible reason for this discrepancy is that the latter used cells that grew a shorter period under long *C* before uploading the baby machine, whereas *W* takes much longer to change, as was the alternative practice [38,39]. This apparent paradox should be thoroughly looked at again to be resolved. A relationship between these two parameters suggests some sort of coupling between their functions, one that is still to be deciphered in molecular terms or otherwise. In the Discussion of Helmstetter’s lecture at the 1968 Cold Spring Harbor Symposium (page 822 in [10]), Dr. GA Herrick suggested that the proportionality between *C* and *D* at slow growth rates “supports the implication of membranes in DNA synthesis”, an interesting idea that has not been pursued yet, to the best of my knowledge.

## 4. Are DNA Functions Exhausted?—Bacterial Cell Dimensions

DNA may have been considered as a reservoir of bases due to its monotonic structure. It was recognized as The Essence Molecule of Life only in the mid-20th century [41]. Its essential roles are still not fully clear. The following fundamental functions of DNA have gradually been discovered over 150 years: chromosome-linked genes, store of genetic information, self-replication, coding for proteins, and the regulation of gene expression. Some others are still under intense investigations, e.g., in bacteria: nucleoid structure, segregation, and “vetoing” cell division (i.e., “nucleoid occlusion”), and involvement in the determination of cell dimensions. I am curious to identify what may be the primary signal(s) for the determination of both cell division and dimensions. Such a signal(s) may turn out to be another function of DNA that would be contained, historically, as “a paradox” in “The Dogmatic Phase” at which we are, as described by Stent [42].

Numerous proteins are jointly and coordinately involved in these crucially essential processes, considered to be downstream of a presumed major signal. Is it an elusive signal that stems from another discipline? Inspired by the “Enzyme-Cannot-Make-Enzyme paradox” [41], I recently proposed a “regulator-of-the-regulator paradox” [43]: “The template feature came from another discipline (information science) than chemistry (producing an enzyme); by analogy, triggering cell division may stem from physics—or another discipline that we are not aware of currently rather than the proteins involved in the division process itself. Can the divisome activation be triggered by the nucleoid’s complexity or replication status?” Two plausible, potential mechanisms related to DNA are transertion [44,45] and supercoiling; the former has been proposed [46,47] as involved in accurately placing the divisome at mid-cell, whereas the latter [48] has yet to be considered. The antibacterial properties of certain naturally derived drugs have recently been mentioned, but their potential action in affecting cell division per se has not been discussed (see “Sitafloxacin” at [49]). In a recent review [50], hyperstructures formed by combining DNA strand-dependent inheritance of nucleoid-associated proteins (NAP) and topoisomerases are proposed to play a central role during the cell cycle by helping generate daughter cells with different phenotypes (via DNA segregation and cell division) and populations with different, average phenotypes (via different degrees of supercoiling).

Whatever the elusive, primary signal(s) for assigning the divisome to precisely act in time and space will be proposed, a powerful tool to test the idea(s) would be, no doubt, the thymine limitation procedure.

## Figures and Tables

**Table 1:** Cell width *W*, nucleoid complexity *NC* and the ratios between them at different doubling times *τ*. (The *oriC*/*terC* ratio *o/t* and the ratios between them and *W* are shown ^c^ for comparisons).

*τ* (min) ^a^	*W* (μm) ^a^	*NC* ^b^	*W*/*NC*	*oriC*/*terC* ^c^	*W*/(*o/t*) ^c^
22	1.43	2.01	0.711	3.53	0.405
32	1.22	1.60	0.762	2.38	0.513
60	0.93	1.28	0.727	1.59	0.585
98	0.87	1.18	0.737	1.33	0.654
17.14	0.98	2.77	0.354	5.06	0.205
22.22	0.80	2.15	0.376	3.43	0.248
26.67	0.72	1.76	0.384	2.83	0.254
30.77	0.67	1.64	0.389	2.51	0.282
37.50	0.61	1.48	0.396	2.08	0.481
50.85	0.55	1.33	0.402	1.73	0.324
51.28	0.55	1.33	0.402	1.72	0.319

Adapted from [28]. ^a^, data from [6,21] (top 4) with *Salmonella typhimuriun* and [29] (bottom 7) with *Escherichia coli*. Values of *W* for *E. coli* were calculated according to *W* = 0.41 × 2^0.36 × 60/*τ*^ [29]. ^b^, *NC* = (2*^n^* − 1)/(*n* × ln2), and ^c^, the ratio *oriC*/*terC* = 2*^n^* were calculated assuming *C* = 40 min.

## Data Availability

Not applicable.
